# The impact of commercialization on the evaluation of DNA evidence

**DOI:** 10.3389/fgene.2013.00227

**Published:** 2013-11-06

**Authors:** Graham Jackson

**Affiliations:** ^1^Advance Forensic ScienceSt. Andrews, UK; ^2^School of Science, Engineering and Technology, University of Abertay DundeeDundee, UK

**Keywords:** likelihood ratio, hierarchy of issues, evaluation, sub-source, source, activity

## Background

Guidance on designing cost-effective examinations and on interpretation of expert observations has been available since the late 1990s in the form of a model framework called Case Assessment and Interpretation (CAI) (Cook et al., 1998a,b; Jackson and Jones, 2009). The underlying principles of the guidance were subsequently encoded in a published standard written by the Association of Forensic Science Providers (Association of Forensic Science Providers, 2009) and have been incorporated in draft guidance from the European Network of Forensic Science Institutes (ENFSI). The guidance is predicated on a logical approach to the evaluation of evidence, requiring examiners to have an understanding and acceptance of the laws of probability. A key element in such an evaluation is the assessment of a likelihood ratio (LR) as the basis of providing logical, balanced, robust, expert opinion.

In addition to the use of an LR approach, a second notion, that of the hierarchy of issues, is a vital element of evaluation. The hierarchy of issues is a scheme that helps identify the case issue that the expert evidence is addressing and thereby clarifies the contribution that evidence is making to the judicial process (Cook et al., 1998b; Evett et al., 2000; Jackson et al., 2006). Using the hierarchy, case issues are classified as belonging to one of four levels—“offence,” “activity,” “source,” or “sub-source” (Jackson, 2009).

This article discusses how commercialization of forensic services, whilst not posing any immediate threat to an evaluation of an LR for scientific evidence, does pose a risk of misleading evidence being adduced if the issue being addressed by the scientist is at too low a level in the hierarchy of issues.

## The hierarchy and DNA evidence

Most DNA cases are reported at, and therefore help the fact-finder (not the scientist) to address issues at a source or sub-source level. If a DNA-profile from a questioned sample can be attributed with a high degree of confidence to a particular body-fluid stain or material, then the results of DNA-profiling help address the issue of the *source* of the *body-fluid*. However, if the DNA-profile cannot be attributed with confidence to a particular body-fluid, then the DNA results help address only the origin of the DNA, i.e., a *sub-source* issue. In a way analogous to Bayesian networks, consideration of sub-source and source level issues then feed into, and inform, consideration of activity level issues and, ultimately, offence level issues.

In some cases, the probative force of matching DNA-profiles for sub-source and source level issues transfers directly, and largely unchanged, to the probative force at activity level, and possibly also to offence level. As an example, consider a case in which a defendant was being tried on a charge of rape. A DNA-profile had been obtained from semen-bearing vaginal swabs taken from the complainant within a few hours of the incident. The profile was found to match that of the defendant. He denies the allegation and declared that he did not know, and had never met, the complainant. Let us assume that the defense are not challenging the prosecutions contentions that:

1. The complainant had been raped by someone.
2. The semen on the vaginal swabs was that of the offender, whomever that may have been.
3. The DNA-profile obtained from the swabs can be attributed with confidence to the semen on the swabs.

In these circumstances, the probative force, in terms of an LR of the order 1 billion provided by the matching DNA-profiles at sub-source level, translates unchanged to a probative force of 1 billion at offence level. Whilst the scientist should preferably be focusing on activity level, she could report the LR at sub-source, source or activity level and there would be little, if any, risk that the court would be misled about the probative force that the matching DNA-profiles provide at offence level, i.e., 1 billion.

Compare that case with one of a burglary in which a scarf was found at the scene. The occupants of the attacked property say that the scarf was not present when they left the premises and it must therefore be a reasonable, but not certain, assumption that the scarf was left by the burglar(s). The scarf was in a dirty, well-worn condition and it bore one small bloodstain. No other blood was found at the scene. DNA-profiling of material cut from the bloodstain on the scarf gave a weak DNA-profile and that was subsequently found to match a suspect. He had no fixed address but shared various flats and “squats” with a number of vagrants and known criminals, often sharing items of clothing. He denied the burglary and said that he couldn't recall wearing a scarf like the one at the scene but did say that he had occasionally worn scarves in the past but that he was not a habitual wearer. No other DNA-analyses were performed to see what other DNA-profiles were present or, indeed, whether the suspect's profile was also present on other non-bloodstained areas of the scarf. Therefore, there is significant uncertainty that the profile could be attributed to the bloodstain. Let us assume that the scientist in this case evaluated and reported the matching DNA-profiles at sub-source level, i.e., helping to address the sub-source issue of “from whom has the DNA originated?” Given a full, matching profile, the scientist reported the LR of a billion as providing “extremely strong support for a view that the DNA originated from the suspect rather than from an unknown, unrelated person.” Without further explanation by the scientist, or guidance from the prosecution, this “value” at sub-source level could be taken by the court and applied erroneously to the “value” that the matching DNA-profiles provided in addressing the offence level issue of whether the suspect committed the burglary. If the scientist wanted to provide more effective, more balanced and robust help to the court, then she should be evaluating and reporting the matching DNA-profiles at activity level, as required by the AFSP standard and CAI principles.

In this last case, specifying an activity level issue would not be a trivial matter. There were no witnesses to the crime and therefore there are no clear activities that constitute the crime and which relate to the scarf. Perhaps the best that scientist could offer would be to consider an issue of whether the suspect was a habitual wearer of the scarf. A pair of appropriate propositions based on the prosecution and defense positions, and conditioned on the relevant background circumstances of the case, could be defined along the lines of:

H_*P*_—The suspect is a habitual wearer of the scarf.
H_*D*_—The suspect is not a habitual wearer of the scarf; someone else is the habitual wearer.

Of course there are problems with defining what “habitual” means in terms of the length of time and the degree of contact that would be classified as “habitual” but let us assume that these variables had been defined broadly. There is also the issue of whether the scarf had been worn habitually by anyone at all. Again, let us assume it would be accepted that it had been worn in such a way. Given sufficient, reliable knowledge of transfer, persistence and detection of DNA-profiles, and on background levels of DNA-profiles, then the scientist may be able to assign probabilities for her observations given the truth of the competing propositions. The observations should include not only the “match” of the profiles but also the quantity and distribution of DNA across the scarf. However, in this case, there is only the observation of a “match”; there is no information on the quantity or distribution of DNA-profiles across the scarf. The scientist is therefore unable to evaluate robustly an LR at this activity level and, in turn, the court does not have the expert help it requires in order to evaluate properly, at offence level, the DNA evidence that has been provided at sub-source level.

Evaluation at activity level of cases in which there is uncertainty on:

– The attribution of the matching profile to a specific body-fluid.
– The relevance to the offence of the matching profile.
– The background presence of the matching profile.

will inevitably mean that the LR provided by matching DNA-profiles at sub-source level, typically of the order 1 billion, will be reduced, sometimes markedly, when that evidence is evaluated at activity and offence levels. Evett et al. (2002) provide examples of two such cases while Biedermann and Taroni (2011) provide a thorough analysis of the relationships and dependencies of the variables involved.

## Practice in england and wales

From anecdotal evidence, particularly from experts working on behalf of the defense, there appears to be a large number of cases, if not the majority of cases, reported at sub-source level with very powerful LRs. However, a significant number require more sophisticated appraisal at activity level in order that the court is not misled on the probative force of the matching DNA-profiles.

In the English and Welsh jurisdiction, police forces pay private companies for the provision of forensic science services. Essentially, under the terms of contracts between the police and the providers, an evaluation of an LR for activity level propositions is generally more costly than for an evaluation at sub-source level. Even if the police or prosecution realize they need an evaluation at activity level, budgetary considerations may deter a request for such an evaluation. Furthermore, even though the AFSP standard requires the scientist to consider activity level, and to advise the customer of the importance of doing so, there is little evidence that providers are able, or willing, to follow that requirement. This may be because the police have submitted for analysis only a sample, such as a swab or piece of fabric, taken from a larger item, depriving the scientist of vital information on the quantity and distribution on that larger item that is necessary for evaluation at activity level.

Providing an evaluation of an LR only at sub-source or source level deprives the court of important information that, in some case, has a direct bearing on the decision of whether the defendant is guilty.

Arguably, many defendants simply plead guilty in the face of expert reports that contain the acronym “DNA” and the figure “1 billion.” This may be because they truly are guilty, or it may be because their lawyers advise them to do so. Or it may be because, while the defendant is innocent, both the defendant and his/her lawyer do not realize that they can challenge this apparently overwhelming figure and that a proper appraisal of the evidence at a more appropriate level would result in much less powerful probative force.

## Disclaimer

The opinions expressed in this article are the personal views of the author. He does not represent any official organization.
